# A novel basement membrane-related gene signature for predicting prognosis of HNSCC

**DOI:** 10.1097/MD.0000000000041316

**Published:** 2025-01-17

**Authors:** Xia Wang, Zhiming Wang

**Affiliations:** a Department of Stomatology, Shengjing Hospital of China Medical University, Shenyang, China.

**Keywords:** basement membrane (BM), HNSCC, overall survival (OS), riskscore, TCGA

## Abstract

In recent years, a notably heterogeneous malignant tumor, squamous cell carcinoma of the head and neck (HNSCC), has received increasing attention, with no significant improvement in its survival rate. The rapid increase in the number of prognostic models associated with HNSCC has been observed due to its accuracy, which offers crucial clinical benefits. The 10 genes were selected from 222 human genes associated with the basement membrane in the analysis of this article. The gene pool was narrowed through different classifications and intersections, followed by univariate Cox regression analysis. Genes with statistical significance underwent further Least Absolute Shrinkage and Selection Operator (LASSO) regression analysis, resulting in the final selection of 10 genes. The data and images extracted from the Human Protein Atlas database were utilized to confirm the differential expression of the corresponding genes. Multivariate Cox regression analysis was employed to develop a nomogram, and the nomogram was assessed by additional decision curve analysis (DCA). The Gene Expression Omnibus validation set was used to validate the established model. Finally, between the high- and low-risk score groups, Gene Set Enrichment Analysis, immune correlation analysis, and drug sensitivity analysis were conducted in this paper. *ITGA5*, *SPOCK1*, *EVA1C*, *TINAGL1*, *LAMB4*, *ADAMTS1*, *EGFL6*, *GPC2*, *BGN*, and *ITGA2B* were successfully developed as basement membrane-associated risk models. The time-dependent receiver operating characteristic (timeROC) curve illustrated that the risk score prediction accuracy outperformed indicators, which were commonly adopted in clinical practice, consisting of age, stage, gender, T-staging, and N-staging. The 3-year risk score timeROC area under the curve value was 0.679. This model demonstrates a reliable ability to assess the prognosis of HNSCC patients. In addition, the specific potential biomarkers associated with the basement membrane were explored in this research.

## 1. Introduction

The basement membrane (BM) has garnered significant attention in recent years due to its correlation with tumor invasion, providing crucial insights for healthcare professionals. The BM is a persistent and universal extracellular matrix that underpins epithelial development and predominantly encases adjacent organs.^[1]^ The primary constituents are collagen, laminin, and integrin, and new research indicates their potential as biological markers for distant metastasis.^[2]^ The BM transitions from epithelial to mesenchymal, facilitating the dissemination of primary malignancies to other organs via the lymphatic vascular system. Research indicates that genes associated with the BM affect survival rates and treatment responses in individuals with gastric cancer,^[3]^ bladder cancer,^[4]^ breast cancer,^[5]^ prostate cancer,^[6]^ glioma,^[7]^ and neurofibromatosis.^[8]^ Nonetheless, the correlation between BM-related genes and squamous cell carcinoma of the head and neck (HNSCC) remains unclear.

About 90% of head and neck cancers are HNSCC, a very heterogeneous form that can manifest in intricate anatomical regions including the mouth, oropharynx, pharynx, larynx, and sinuses. As to the Global Burden of Cancer data published in 2020, it is estimated that there will be 1.93 million new cancer cases globally (excluding nonmelanoma skin cancers), of which 0.23 million cases will be head and neck cancer.^[9]^ Therefore, it is essential to create prognostic models for more accurate prediction of patient clinical outcomes, thereby aiding doctors in making focused treatment decisions.^[10]^

Clinical factors, tumor histology, and specific altered genes are methods to estimate the overall survival of individuals with HNSCC. Currently, gene-based prognostic models have been thoroughly examined for predicting overall survival in patients with HNSCC in conjunction with other clinical markers.^[11–13]^ The integrity of the BM alters during inflammation, facilitating the ingress of inflammatory cells into the epithelium. The BM structure undergoes alterations following the invasion of cancer cells in tumors. Laminin is the predominant noncollagenous element in all BMs. Cytoplasmic laminin expression levels were elevated in poorly differentiated invasive oral squamous cell carcinoma.^[14]^ A study aims to develop an in vitro 3D model that simulates intraoral HNSCC, with primary advantages including the ability of cancer cells to infiltrate the connective tissue matrix following the rupture of the BM.^[15]^ The BM serves as a critical barrier against cancer cell invasion and significantly influences the survival prognosis of tumor patients. Oral dysplasia and squamous cell carcinoma demonstrate modified signaling pathways associated with cell motility, BM degradation, and metastasis.^[16]^ Nonetheless, comprehensive investigations of BM-associated genes in HNSCC remain insufficient. This study aims to utilize statistical approaches to investigate BM-associated genes in HNSCC. This study also developed a model utilizing prognostic genes associated with the BM and clinical factors to predict overall survival in individuals with HNSCC.

## 2. Materials and methods

### 2.1. Data source

In the beginning, the gene expression data as well as the clinical information of the corresponding sample were downloaded from the Cancer Genome Atlas (TCGA) (https://portal.gdc.cancer.gov/repository) and Gene Expression Omnibus (https://www.ncbi.nlm.nih.gov/geo/) databases, while survival information, which is from the same sample as the first 2, was downloaded from the UCSC Xena database up to September 20, 2022 (http://xena.ucsc.edu/). GSE65858 served as a validation set, which is similar to the previous study.^[11]^ Furthermore, 222 BM-related genes were validated and analyzed in our study.^[1]^

### 2.2. Identify differential genes associated with BMs

In order to illustrate the expression levels of BM-associated genes, the TCGA-HNSCC data set, which is composed of 515 tumor tissues and 44 normal tissue samples, was employed in this paper. The R package “DESeq2” within the R software was adopted for differential analysis in the TCGA-HNSCC cohort. Genes shared between differentially expressed genes (DEGs) and BM-related genes were chosen for further investigation and visualized via heat map and volcano map.^[17]^ Furthermore, the protein-protein interaction network for investigating the interactions among the BM-related DEGs was constructed by leveraging the Search Tool for Retrieval of Interacting Genes/Proteins database (http://www.string-db.org/).

### 2.3. Develop a risk model related to the BM

The TCGA-HNSCC data set is a training set that was adopted for the development of the risk model in this research. Using univariate Cox analysis, we looked for DEGs connected to the BM that had a big effect on the overall survival rate in the TCGA-HNSCC group at a 5% level of significance. A R package named glmnet as well as lasso-penalized Cox regression analysis were utilized to minimize overfitting in the risk model. The risk score is calculated as follows: $\underset{i=1}{\overset{n}{\sum }}\;(Exp_{i}\times Coe_{i})$. Where, $Exp_{i}$ represents expression level of BM-related DEGs as well as $Coe_{i}$ represents regression coefficient of the corresponding genes. On account of the median risk score, patients diagnosed with HNSCC are categorized into high-risk and low-risk groups. Additionally, univariate and multivariate Cox regression analyses were employed to assess the prognostic value, which is independent of each other of the model consisting of 10 genes.

### 2.4. Validate the BM-related risk model

The Gene Expression Omnibus data set (GSE65858) was a validation set for validating the risk model, and the identical risk score calculation formula as described above was adopted in this verification set. Taking advantage of the R package named Survival, survival analysis was conducted, and Kaplan-Meier curves were generated. R package “pheatmap” was employed to plot the risk score curve. The time-dependent receiver operating characteristic (timeROC) method was used to predict the sensitivity and specificity of the predictive power of the risk model. On account of the risk score, age, gender, and stage, the R package “rms” was employed to create a nomogram in patients diagnosed with HNSCC. Additionally, in order to evaluate the accuracy of the nomogram, decision curve analysis and timeROC were employed in this study.

### 2.5. Gene enrichment analysis and immunoassay

The R package “clusterProfiler” was utilized to examine the potential function of candidate genes. Gene Set Enrichment Analysis (GSEA) revealed that the gene signaling pathway was significantly enriched (p≤0.05,q≤0.05). Additionally, using the single-sample GSEA function available in the “GSVA” R package, the infiltration scores of 28 immune cells were examined. By employing Cell Infiltration By Recursive Partitioning (CIBERSORT), an immunoassay was conducted to evaluate the degree of immune cell infiltration in various samples. For CIBERSORT, the number of iterations was set to 100. The calculation yielded a result in which *P* values >0.05 were discarded. Using the Estimation of STromal and Immune cells in MAlignant Tumour tissues using Expression data (ESTIMATE) algorithm, stromalScore, tumorPurity, and ESTIMATEScore, which were 3 indicators of the tumor microenvironment status, were calculated.

### 2.6. Drug sensitivity

The “oncoPredict” software package within the R programming language was utilized to determine drug sensitivity. Between high- and low-risk groups, a significant difference was observed in drug sensitivity (*P* ≤ .001). The medications analyzed for sensitivity were either Food and Drug Administration-approved or undergoing clinical trials.

### 2.7. Statistical analysis

All statistical computations were executed utilizing R software (version 4.3.2). Within the TCGA pipeline, the “DESeq2” package, which is based on R software, was employed to identify DEGs. The criteria for identifying DEGs are set as: p≤0.05&|Foldchange|≥1. Survival analysis was conducted between both risk groups using the R package “Survival.” We used the “timeROC” package in R and both univariate and multivariate Cox regression analyses to find out how useful risk scores and related clinical indicators are for predicting the future. The predictive potential of the model was evaluated using the AUC of the timeROC curve. The Wilcoxon test was applied to analyze the box plots of the 2 groups. In order to obtain *P* values and hazard ratios in Kaplan-Meier survival analysis, the log-rank test and univariate Cox proportional hazard regression were adopted in this study. A 2-sided *P*-value <0.05 was deemed statistically significant.

## 3. Result

### 3.1. Identification of BM-related DEGs

**Figure 1.**
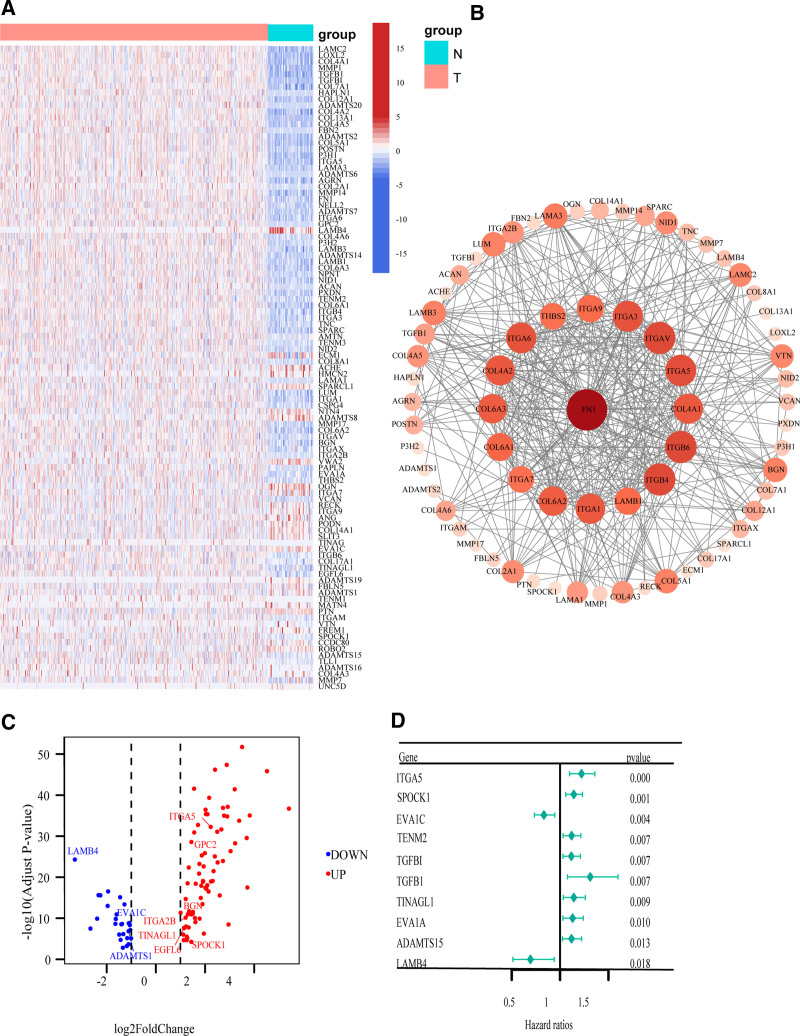
Identification of basement membrane-related DEGs. (A) Heat map showing the expression of 103 basement membrane-related DEGs. (B) Interaction network of 103 basement membrane-related DEGs. (C) Volcano plot of 103 basement membrane-related DEGs. Red nodes represent upregulated genes with logFC ≥ 1 and *P* ≤ .05. (D) Univariate cox regression analysis of 10 prognostic basement membrane-related genes. DEG= differentially expressed genes, logFC = log2-based fold change.

A total of 559 samples were gathered within the TCGA-HNSCC cohort, which comprised 515 tumor tissues and 44 normal tissues. In our research, based on the criteria that p≤0.05&|Foldchange|≥1 4810 differential genes were identified. Out of these, the expression levels of 2414 genes were significantly upregulated, while 2396 genes demonstrated significantly downregulated expression. The intersection of the DEGs with 222 genes associated with the human BM identified 103 differential genes, among which 75 genes were upregulated (Fig. 1A and C).

**Figure 2.**
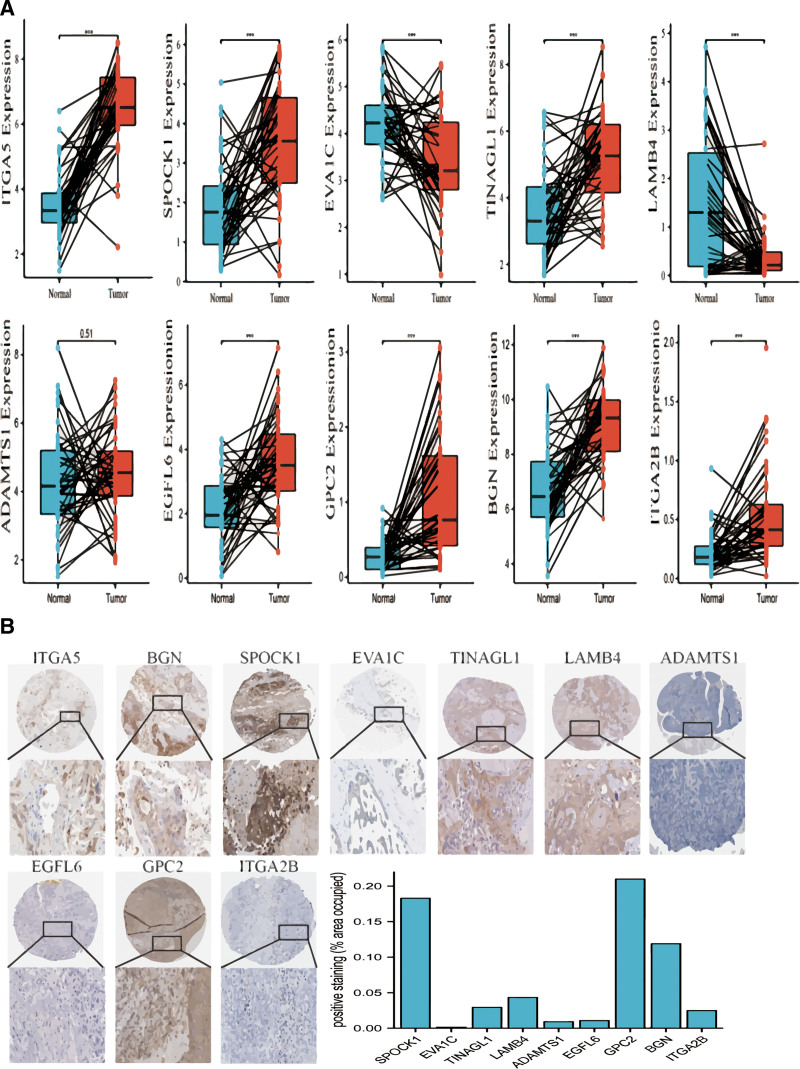
The expression levels of 10 basement membrane-associated differentially expressed genes (DEGs). (A) The mRNA expression of ten basement membrane-associated DEGs. (B) The protein expression of ten basement membrane-associated DEGs in the Human Protein Atlas database. ***P < .001; **P < .01; *P < .05.

The protein-protein interaction network illustrates the degree of number of associated nodes about basal membrane-associated DEGs (Fig. 1B). More univariate Cox regression analysis showed that 21 DEGs associated with the basal membrane were linked to the prognosis of HNSCC patients. These 21 genes were divided into 2 groups: risk genes (16 were *ITGA5*, *SPOCK1*, *TENM2*, *TGFBI*, *TGFB1*, *TINAGL1*, *EVA1A*, *ADAMTS15*, *ITGA3*, *MMP14*, *ADAMTS1*, *LAMC2*, *LAMB3*, *ITGA6*, *FN1*, and *BGN*) and protective genes (5 were *EVA1C*, *LAMB4*, *EGFL6*, *GPC2*, and *ITGA2B*). We chose the top 10 genes with the most significant *P* values and presented them in a forest map format (Fig. 1D). Later on, the LASSO-Cox regression analysis of these genes was carried out, and 10 candidate prognostic genes (*ITGA5*, *SPOCK1*, *EVA1C*, *TINAGL1*, *LAMB4*, *ADAMTS1*, *EGFL6*, *GPC2*, *BGN*, and *ITGA2B*) were ultimately selected. The expression level analysis of paired samples showed that these genes (*ITGA5*, *SPOCK1*, *TINAGL1*, *EGFL6*, *GPC2*, *BGN*, and *ITGA2B*) were significantly higher in people with HNSCC. In contrast, the expression of *EVA1C* and *LAMB4* showed a downward trend, while *ADAMTS1* exhibited no significant difference (Fig. 2A). In order to further explore the expression of these 10 genes, a bar chart was depicted using the Human Protein Atlas database (Fig. 2B).

**Figure 3.**
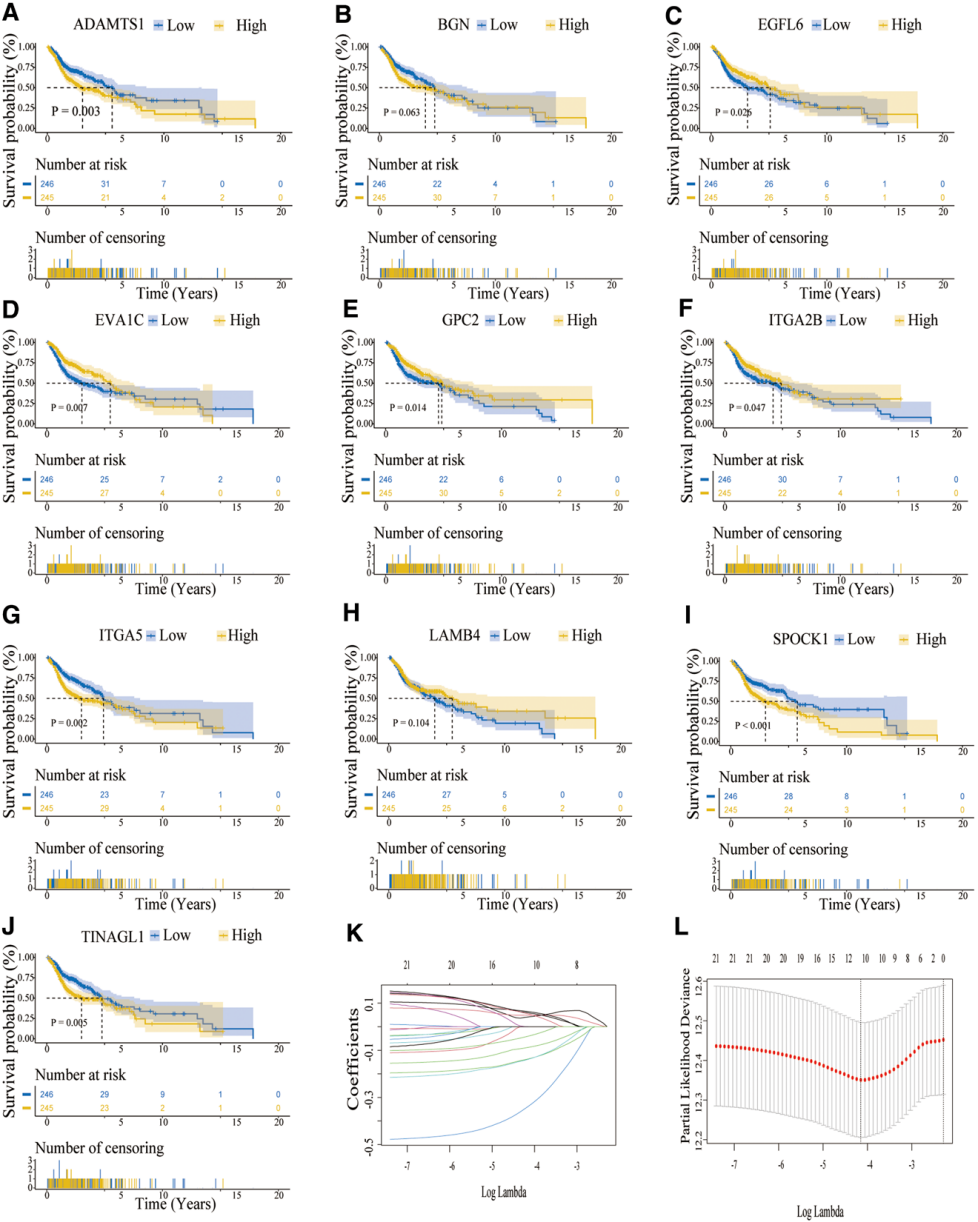
The survival analysis of ten hub basement membrane-associated genes and LASSO analysis. (A–J) The survival probability of *ADAMTS1* (A), *BGN* (B), *EGFL6* (C), *EVA1C* (D), *GPC2* (E), *ITGA2B* (F), *ITGA5* (G), *LAMB4* (H), *SPOCK1* (I), and *TINAGL1* (J). (K) LASSO coefficients profiles of basement membrane-associated differentially expressed genes (DEGs). (L) LASSO-penalized regression with 10-fold cross-validation obtained 10 genes using minimum lambda value. LASSO = Least Absolute Shrinkage and Selection Operator.

Survival analysis revealed that a low expression of *ADAMTS1*, *ITGA5*, *SPOCK1*, and *TINAGL1* was associated with improved prognosis (Fig. 3A, G, I, and J). On the contrary, *EGFL6*, *EVA1C*, *GPC2*, and *ITGA2B* showed the opposite trend (Fig. 3C–F). The impact of *BGN* and *LAMB4* on the overall survival of HNSCC patients was not statistically significant (Fig. 3B and H). These findings suggest that BM-associated DEGs might be involved in the progression of HNSCC.

### 3.2. Building a BM-related risk model

**Figure 4.**
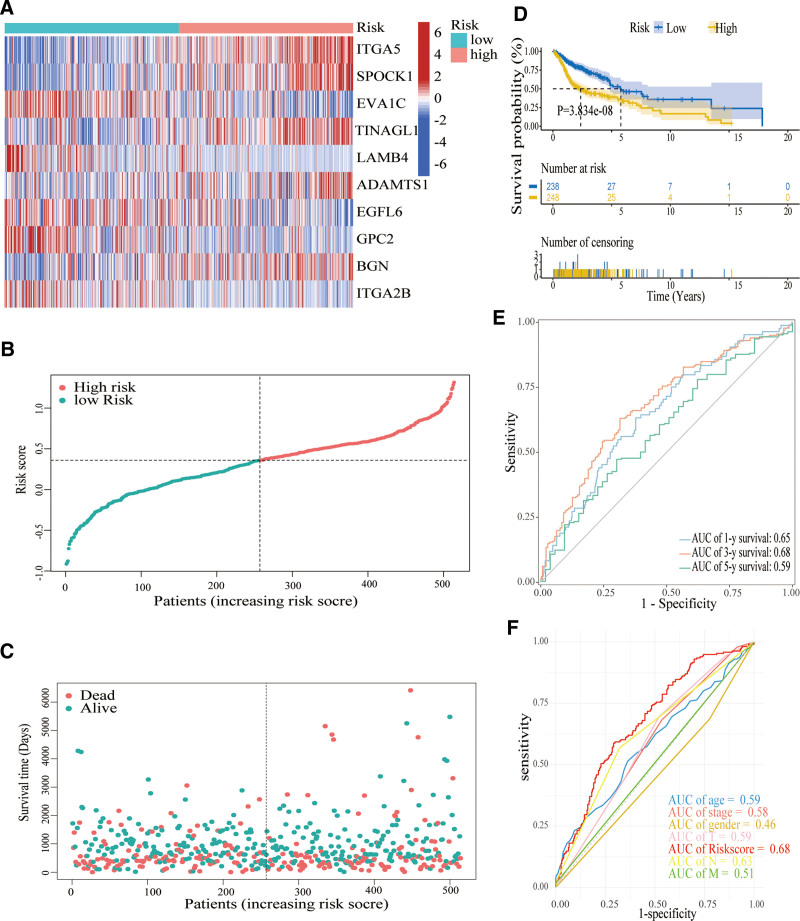
Prognostic value of 10 gene signature in TCGA-HNSCC cohort. (A) Heat map of these 10 genes between the 2 risk groups. (B) The line connected by dots represents the median risk score, which divides patients diagnosed with HNSCC into high-risk and low-risk groups. (C) Distribution of survival status between the 2 risk groups. Patients with positive survival outcomes had higher risk scores. (D) Kaplan-Meier survival analysis. (E) TimeROC analysis of the 10-gene signature. (F) AUC of Riskscore and clinical traits. AUC = area under the curve, HNSCC = squamous cell carcinoma of the head and neck, TCGA = Cancer Genome Atlas.

A LASSO-penalized Cox regression analysis was used to create a risk model for HNSCC patients based on their BMs while minimizing the risk of overfitting. The LASSO algorithm was used for candidate prognostic DEG selection and shrinkage with the “glmnet” R package. The response variables were overall survival and the status of patients in the TCGA cohort. The penalty parameter (λ) for the model was determined by 10-fold cross-validation following the minimum criteria (ie, the value of *λ* corresponding to the lowest partial likelihood deviance) (Fig. 3K–L). Finally, we screened 10 candidate prognostic genes related to the BM. The risk scores of the patients were calculated according to the normalized expression level of each gene and its corresponding regression coefficients. Just as score = esum (each gene’s regression coefficient × corresponding expression). In this study, the calculation of the risk model is as follows: (0.0413 × *ITGA5*) + (0.0489 × *SPOCK1*) + (−0.0878 × *EVA1C*) + (0.0590 × *TINAGL1*) + (−0.3380 × *LAMB4*) + (0.0640 × *ADAMTS1*) + (−0.1367 × *EGFL6*) + (−0.1383 × *GPC2*) + (0.0542 × *BGN*) + (−0.0402 × *ITGA2B*). As illustrated in the heat map, variations in the expression of these 10 genes were observed between the 2 risk groups (Fig. 4A). The distribution of risk scores as well as survival status was depicted in the TCGA-HNSCC patient cohort (Fig. 4B and C).

Notably, compared with the high-risk group (n = 248), the low-risk group (n = 238) has a superior overall survival (*P* < .001; Fig. 4D). In order to assess the predictive veracity of the risk score, the timeROC method was conducted and yielded 1-, 3-, and 5-year AUC values of 0.65, 0.68, and 0.59, respectively. In comparison to clinical indicators consisting of grade, T, N, M, stage, age, and sex, the AUC value of the risk score stood at 0.68, contrasting with 0.58, 0.59, 0.63, 0.51, 0.59, and 0.46. These results suggest that the risk score was an excellent biomarker, which was independent of each other for HNSCC prognosis (Fig. 4E and F).

### 3.3. Verifying the BM-related risk signature

**Figure 5.**
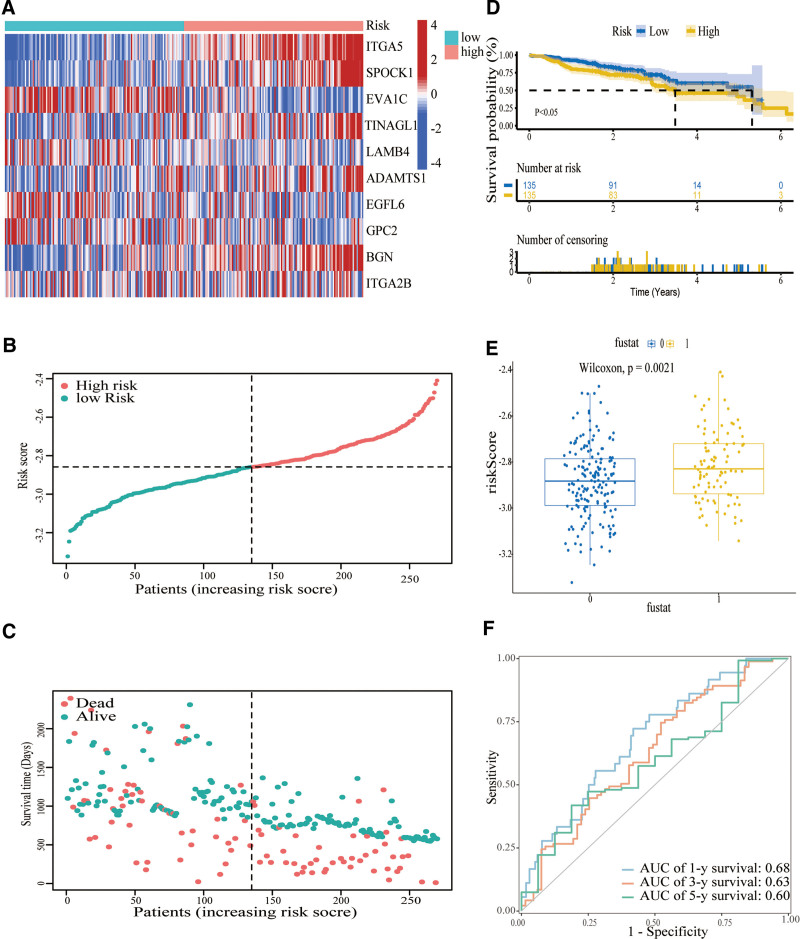
Prognostic research of ten gene signature in GSE65858 cohort. (A) In the 2 risk groups, the heat map of the 10 genes. (B) A curve of risk score. (C) Distribution of the survival status of the 2 risk groups. (D) Kaplan-Meier survival analysis in the 2 risk groups. (E) The box diagram shows the risk scores for different survival states. (F) TimeROC analysis of the signature consisting of 10 gene. AUC = area under the curve.

Using the GSE65858 (n = 270) data set, the signature was validated in order to further assess the risk signature associated with the BM. Ten genes related to the BM showed differential distribution between the 2 risk groups (Fig. 5A) as well as the group of HNSCC (n = 135) with a high-risk score, which demonstrated an earlier probability of mortality (Fig. 5B and C) and presented an inferior OS (*P* < .05; Fig. 5D). Patients with HNSCC who experienced mortality had significantly higher risk scores than those who survived at the significance level of 0.2% (Fig. 5E). TimeROC curve analysis revealed the AUC values for 1, 3, and 5 years were 0.68, 0.63, and 0.60, respectively (Fig. 5F).

### 3.4. Prognostic value of BM-related risk model

**Figure 6.**
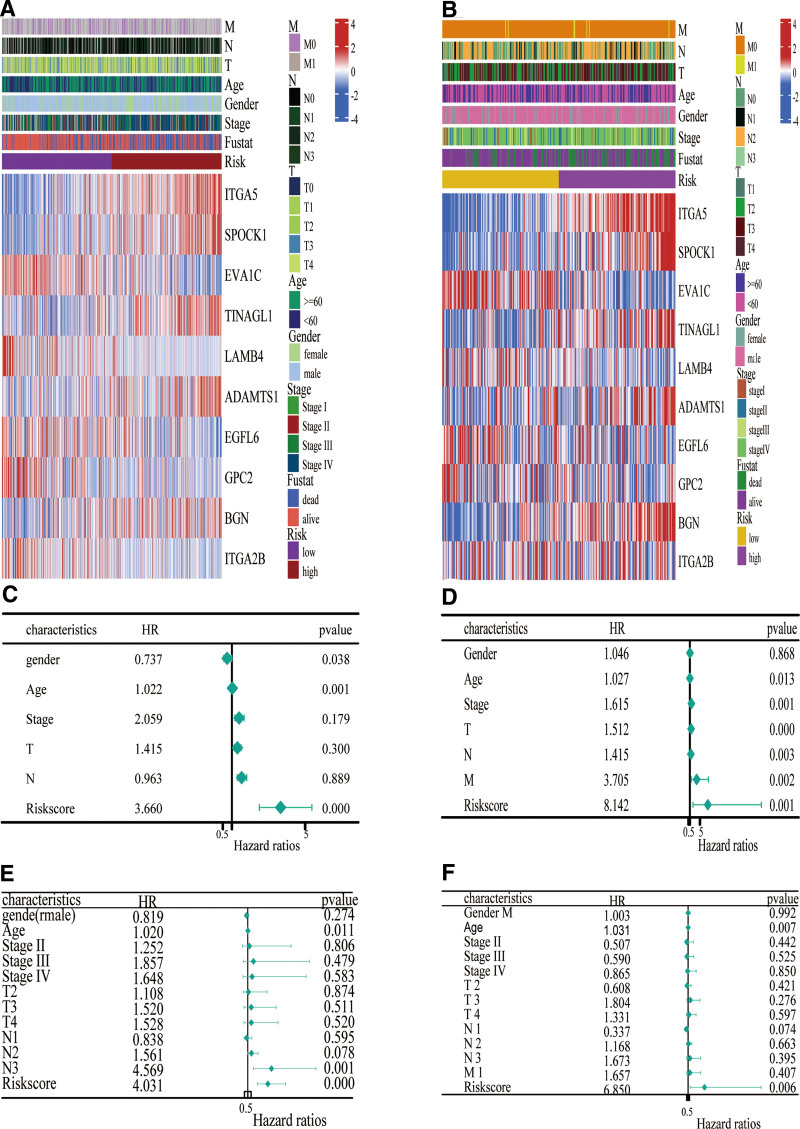
Prognostic value of the 10-gene signature. (A) Heat map of clustering of 10 genes in TCGA-HNSCC. (B) Heat map of clustering of ten genes in GSE65858. (C) Univariate Cox analysis of clinical characteristics as well as risk score in the TCGA-HNSCC cohort. (D) Univariate Cox analysis of clinical characteristics as well as risk score in the GSE65858 cohort. (E) Multivariate Cox analysis in the TCGA-HNSCC cohort. (F) Multivariate Cox analysis in the GSE65858 cohort. HNSCC = squamous cell carcinoma of the head and neck, HR = hazard ratio, TCGA = Cancer Genome Atlas.

In order to investigate whether BM-related risk scores can serve as prognostic factors that were independent of each other, the distinctions between the 2 risk groups were further examined (Fig. 6A and B), and BM-related genes exhibited a significant distribution pattern. Using the TCGA-HNSCC as well as GSE65858 cohorts, for comparing the BM risk scores with clinical markers, Cox regression analysis was employed. It can be observed from the univariate Cox regression analysis that gender (*P* = .038), age (*P* = .001), and risk score (hazard ratio [HR] = 3.660; *P* < .001) were found to be significant upon survival (Fig. 6C). Age (*P* = .011), N3 versus N0 in the N stage (*P* = .001), and risk score (HR = 4.031; *P* < .001) were deemed as crucial factors for OS in the multivariate Cox regression (Fig. 6E). Similar findings were observed in the GSE65858 data set. In the univariate Cox analysis, the risk score emerged as a significant predictor of HNSCC prognosis (HR = 8.142, *P* = .001), and in the multivariate Cox analysis, it continued to serve as a crucial predictor of HNSCC prognosis (HR = 6.850, *P* = .006, Fig. 6D and F). Therefore, the BM-related risk score is an independent predictor of HNSCC prognosis.

### 3.5. Construct and validate a nomogram based on BM-associated DEGs

**Figure 7.**
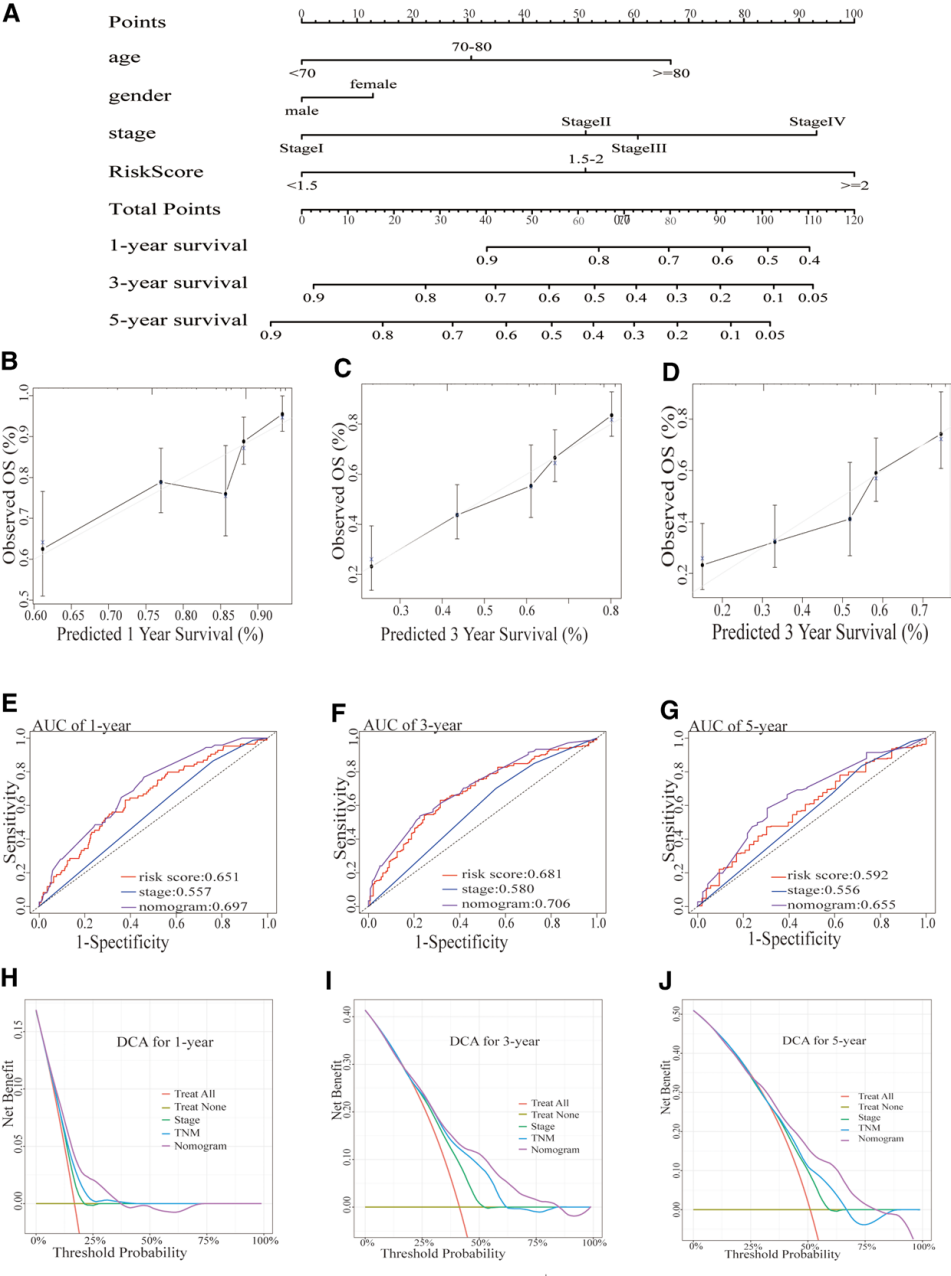
Construct and validate the nomogram based on basement membrane-associated DEGs in TCGA data set. (A) Nomogram originated in risk score as well as clinical markers. (B–D) Calibration plot of the nomogram. (E–G) TimeROC curves of the nomogram. (H–J) A DCA based on the nomogram. AUC = area under the curve, DCA = decision curve analysis, DEG = differentially expressed gene, OS = overall survival, TCGA = Cancer Genome Atlas.

In order to predict 1-, 3-, and 5-year OS rates of HNSCC patients by integrating 10 prognostic human BM DEGs with additional clinical markers consisting of age as well as pathological stage, a nomogram was constructed. Owing to the precision of the multifactor Cox proportional hazards model, we have established age, pathological stage, and risk score as multiple categorical variables for the construction of this nomogram (Fig. 7A). The nomogram demonstrated a satisfactory concurrence with the actual outcomes (Fig. 7B-D). Given the limited number of patients with stage M1 in the TCGA-HNSCC cohort, only 1 patient was available. Therefore, we omitted the M stage when constructing the commonly employed TNM staging model in clinical practice. However, the model was still referred to as TNM in accordance with convention. The timeROC curve revealed that the accuracy of the nomogram model exceeded that of the pathological stage model and risk scoring model (Fig. 7E–G). The timeROC AUC values for the 1-, 3-, and 5-year nomograms were 0.697, 0.706, and 0.655, separately. It can be observed that when the threshold probability is set above 0.2, decision curve analysis indicates that the nomogram yields more favorable outcomes than the stage as well as TNM models (Fig. 7H–J).

### 3.6. GSEA as well as immunoassay

**Figure 8.**
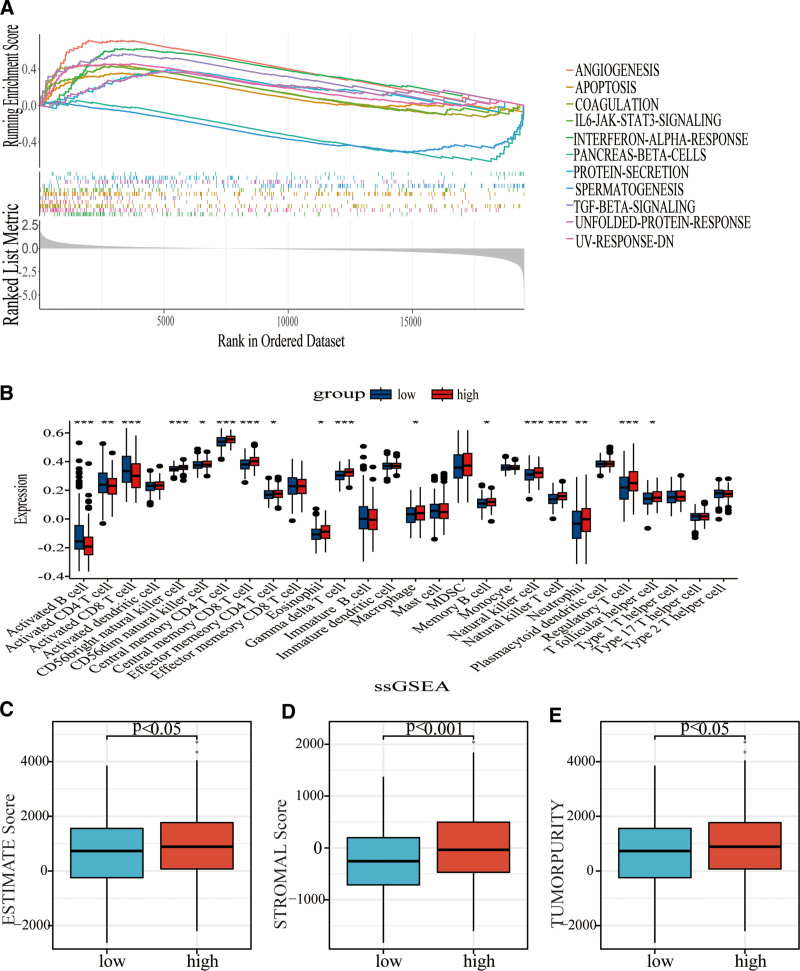
GSEA and immunoassay of basement membrane-related signature. (A) GSEA analysis between the 2 risk groups. (B) The ssGSEA analysis. (C–E) The estimate score (C), stromal score (D), and tumor purity (E) between ESTIMATE algorithms. GSEA = Gene Set Enrichment Analysis, ESTIMATE = Estimation of STromal and Immune cells in MAlignant Tumour tissues using Expression data, ssGSEA = single sample Gene Set Enrichment Analysis.

**Figure 9.**
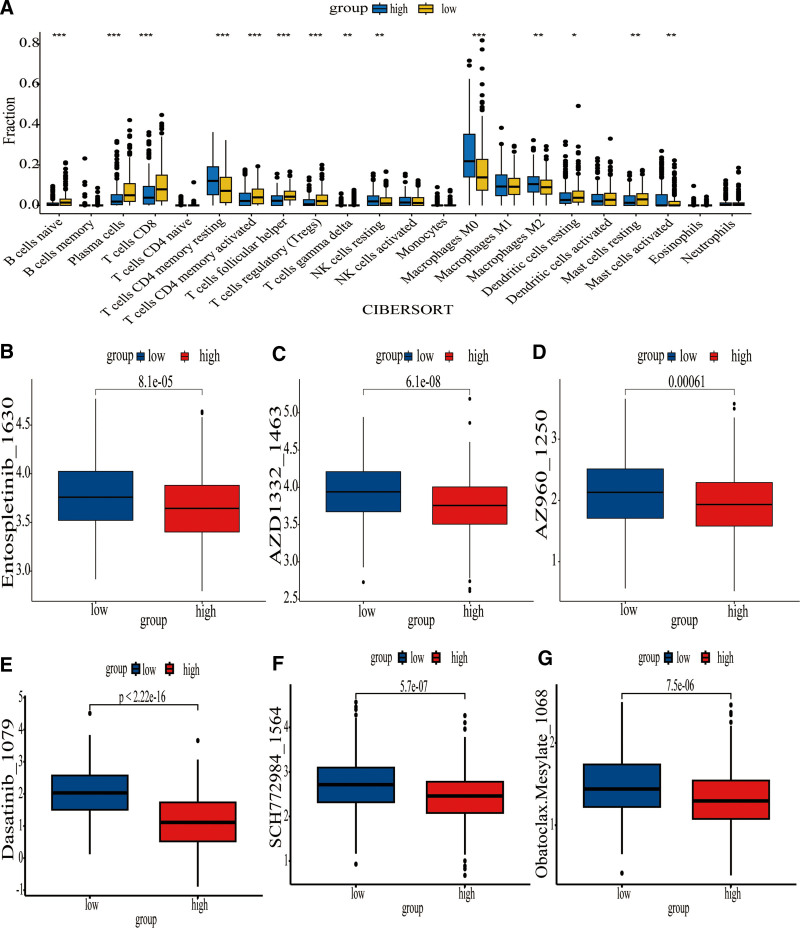
CIBERSORT as well as drug sensitivity analysis between the 2 risk groups. (A) The estimation of relative abundance of immune cells. (B–G) The drug sensitivity to Entospletinib_1630 (B), AZD1332_1463 (C), AZ960_1250 (D), Dasatinib_1079 (E), SCH772984_1564 (F), and Obatoclax.Mesylate_1068 (G). CIBERSORT = Cell Infiltration By Recursive Partitioning.

To elucidate the role of BM-related candidate genes, GSEA was employed to investigate potential signaling pathways. The results indicated that these genes were connected with some signaling pathways, including gene variation, autophagy, agglutination, IL6_JAK_STAT3, and α-interferon response at the 5% significance level (Fig. 8A). It can be observed from Figure 8B that single-sample GSEA analysis revealed that with a higher risk score, the immune cell abundance consisting of activated B cells as well as activated CD4 T cells was lower (*P* < .05). The ESTIMATE algorithm determined that the high-risk group demonstrated no significant difference in ImmuneScore but had a higher StromalScore at the significance level of 0.1% and tumor purity (*P* < .05; Fig. 8C–E). A similar result was observed, as illustrated in the CIBERSORT; with a higher risk score, the relative abundance of immune cells was lower (Fig. 9A). Together, these findings provide compelling evidence that these DEGs associated with the BM might be involved in HNSCC progression by regulating the infiltration of immune cells in tumor microenvironment.

### 3.7. Drug sensitivity analysis

A further comparison can be made between the 2 risk groups, and in order to identify potential drugs targeting BM-associated genes, drug sensitivity analyses were conducted. The findings revealed that the low-risk group’s sensitivity to Dasatinib_1079, Entospletinib_1630, AZD1332_1463, AZ960_1250, SCH772984_1564, and Obatoclax.Mesylate_1068 was significantly higher than the high-risk groups at the 0.1% significance level (Fig. 9B–G). As a result, genes related to the BM could be used as molecular targets to improve cancer chemotherapy, targeted therapy, and immune system therapies.

## 4. Discussion

HNSCC is a serious cancer impacting the human body. Reports suggest that new instances of head and neck cancer will represent 11.80% of all newly diagnosed malignant tumors in 2020.^[9]^ Therefore, it is essential to find viable biomarkers and establish a suitable signature to predict the overall survival rate of patients diagnosed with HNSCC. Most prior research determined the survival prediction for individuals with HNSCC exclusively on clinical indicators or a singular gene.^[18–20]^ Despite the prevalent use of the TNM staging system in clinical practice, as noted in the introduction, both clinical parameters and gene expression levels demonstrate restricted prognostic capability. Therefore, improving prediction accuracy requires augmenting the number of predictive parameters. In the past 10 years, the molecular genetic framework of HNSCC has been clarified, revealing novel avenues for therapeutic intervention. Current research endeavors aim to enhance the development of therapies that maximize efficacy and minimize adverse effects by acquiring and synthesizing knowledge about HNSCC biology and immunology to identify predicted biomarkers.^[21]^

The BM serves as an anatomical barrier to invasive malignancy. Invasive HNSCC complicates radical treatment due to its involvement with local and lymphatic vessels. Invasive HNSCC necessitates tumor cells to eliminate spatial limitations on growth and spread by gaining the capacity to destroy the BM and lamina propria extracellular matrix.^[22]^ The composition and regulatory roles of the BM are yet to be clarified. Laminin, an essential constituent of the BM, plays a pivotal role in angiogenesis, cellular migration, and adhesion. The *LAMB4* gene, which encodes a laminin chain, has been linked to the onset of gastric and colorectal cancers.^[23]^ Huang et al^[24]^ previously indicated that the silencing of *LAMB4* in HNSCC cell lines could enhance cell proliferation and migration. The *BGN*/*PF-STAT3* positive feedback loop can promote peritoneal metastasis in gastric cancer patients,^[25]^ and *BGN* is associated with the degree of immune cell infiltration.^[26]^ Zhao et al^[27]^ indicated that *BGN* has markedly elevated expression in individuals with HNSCC and correlates with reduced overall survival. *ITGA5* facilitates tumor angiogenesis in cervical carcinoma.^[28]^ Clinical data analysis reveals that the activation of the mTORC1-*ITGA5*-*EFNB2* signaling pathway is associated with malignant development and poor prognosis in laryngeal cancer patients.^[29]^ In HNSCC, *ACTN1* interacts with *ITGA5* to promote cell proliferation, invasion, and epithelial-mesenchymal transition.^[30]^ Prior research has demonstrated that *SPOCK1* is linked to cancer in various tissues, including the ovaries,^[31]^ lungs,^[32]^ liver,^[33]^ and breasts.^[34]^ Research utilizing si-*SPOCK1* knockdown has shown that elevated *SPOCK1* expression promotes the aggressiveness of HNSCC cells.^[35]^ Wang et al^[36]^ observed that elevated levels of ITGA2B correlate with enhanced outcomes in patients with HNSCC, a conclusion that aligns with our findings (see Fig. 3F). However, *EVA1C*, *TINAGL1*, *ADAMTS1*, *EGFL6*, and *GPC2*, which correlate with overall survival in HNSCC patients, have not been documented or evaluated concerning HNSCC. Our data indicate that these 5 genes may represent novel treatment targets for HNSCC, and their mechanisms of action require further investigation.

The current research indicates a low overall survival rate associated with a greater risk score, which demonstrates superior predictive accuracy for survival prognosis compared to other clinical indicators. Drug sensitivity studies revealed that individuals with elevated risk scores exhibited a reduced likelihood of responding to these chemotherapeutic agents. Collectively, these findings offer persuasive evidence that the BM-associated risk signature may not only forecast prognosis but also enhance therapeutic practice. Nonetheless, this research is subject to specific limitations: on one side, all data utilized in our study was sourced from public sources, which do not include complete HNSCC cases or the associated mutations. Conversely, HNSCC has considerable variety, rendering risk assessment inadequate for encompassing various clinical kinds. Therefore, additional investigation is necessary, attainable through the integration of single-cell RNA sequencing and spatial transcriptome analysis. Furthermore, although these 10 genes associated with HNSCC were statistically excluded and their mechanisms investigated, this study lacked experimental validation, hence providing a basis for our future research.

## 5. Conclusion

In summary, genes linked to the BM were incorporated into our prognostic model research. Additionally, the prognostic condition, immune cell infiltration, and medication sensitivity of HNSCC patients were evaluated based on risk scores. This research could gain from a more profound comprehension of the importance of BM-associated genes and the identification of new treatment targets for HNSCC.

## Author contributions

**Data curation:** Xia Wang.

**Formal analysis:** Xia Wang.

**Investigation:** Xia Wang.

**Software:** Xia Wang.

**Visualization:** Xia Wang.

**Writing – original draft:** Xia Wang.

**Writing – review & editing:** Xia Wang, Zhiming Wang.

**Supervision:** Zhiming Wang.
